# Consumption of fruits and vegetables among older adults: findings from the ELSI‐Brazil study

**DOI:** 10.1590/0102-311XEN158122

**Published:** 2023-07-17

**Authors:** Paula Bolbinski, Mary Anne Nascimento-Souza, Maria Fernanda Lima-Costa, Sérgio Viana Peixoto

**Affiliations:** 1 Instituto René Rachou, Fundação Oswaldo Cruz, Belo Horizonte, Brasil.; 2 Programa de Pós-graduação em Saúde Púbica, Universidade Federal de Minas Gerais, Belo Horizonte, Brasil.; 3 Escola de Enfermagem, Universidade Federal de Minas Gerais, Belo Horizonte, Brasil

**Keywords:** Aging, Eating, Sociodemographic Factors, Life Style, Envelhecimento, Ingestão de Alimentos, Fatores Sociodemográficos, Estilo de Vida, Envejecimiento, Ingestión de Alimentos, Factores Sociodemográficos, Estilo de Vida

## Abstract

This study aimed to assess the prevalence of recommended consumption of fruits and vegetables and their associated factors in a national sample representative of the Brazilian population aged 60 or over. Baseline data from the *Brazilian Longitudinal Study of Aging* (ELSI-Brazil), conducted from 2015 to 2016, including 4,982 older individuals, were used. The recommended consumption of fruits and vegetables was assessed based on questions on the weekly and daily frequency of fruits, natural fruit juice, and vegetables. Intake of five or more servings of these foods on five or more days per week was considered as recommended consumption. Exploratory variables included socio-demographic characteristics, health behaviors, health conditions, and use of health services. Univariate and multiple logistic regression were used to examine the factors associated with the recommended consumption of fruits and vegetables. The prevalence of recommended consumption of fruits and vegetables was 12.9% (95%CI: 11.5-14.3). This consumption showed associations with gender (women - OR = 1.40; 95%CI: 1.08-1.82), age group (80 years or older - OR = 1.66; 95%CI: 1.16-2.37), education level (8 years or more - OR = 2.07; 95%CI: 1.51-2.86), smoking (former smokers - OR = 0.69; 95%CI: 0.55-0.85 and current smokers - OR = 0.50; 95%CI: 0.33-0.77) and medical appointments in the previous 12 months (OR = 1.88; 95%CI: 1.31-2.71). Our findings showed a low prevalence of the recommended consumption of fruits and vegetables among older Brazilian adults, drawing attention to the need for policies aimed at increasing this consumption in the studied population.

## Resumo

O objetivo deste estudo foi avaliar a prevalência do consumo recomendado de frutas e hortaliças e seus fatores associados em uma amostra nacional representativa da população brasileira com 60 anos ou mais. Foram utilizados dados da linha de base do *Estudo Longitudinal da Saúde dos Idosos Brasileiros* (ELSI-Brasil), realizado entre 2015 e 2016 com 4.982 idosos. O consumo recomendado de frutas e hortaliças foi avaliado com base em questões sobre a frequência semanal e diária de frutas, sucos naturais e hortaliças. A ingestão de cinco ou mais porções desses alimentos em cinco ou mais dias por semana foi considerada como consumo recomendado. As variáveis exploratórias incluíram características sociodemográficas, comportamentos de saúde, condições de saúde e o uso de serviços de saúde. A regressão logística univariada e múltipla foi utilizada para examinar os fatores associados ao consumo recomendado de frutas e hortaliças. A prevalência do consumo recomendado de frutas e hortaliças foi de 12,9% (IC95%: 11,5-14,3). Esse consumo apresentou associação com sexo (feminino - OR = 1,40; IC95%: 1,08-1,82), faixa etária (80 anos ou mais - OR = 1,66; IC95%: 1,16-2,37), escolaridade (8 anos ou mais - OR = 2,07; IC95%: 1,51-2,86), tabagismo (ex-fumantes - OR = 0,69; IC95%: 0,55-0,85; e fumantes - OR = 0,50; IC95%: 0,33-0,77) e número de consultas médicas nos últimos 12 meses (OR = 1,88; IC95%: 1,31-2,71). Os resultados deste estudo mostraram uma baixa prevalência do consumo recomendado de frutas e hortaliças entre idosos brasileiros, chamando a atenção para a necessidade de políticas voltadas para o aumento desse consumo na população estudada.

## Resumen

El objetivo de este estudio fue evaluar la prevalencia del consumo recomendado de frutas y verduras y sus factores asociados en una muestra nacional representativa de la población brasileña con 60 años o más. Se utilizaron datos de línea de base del *Estudio Longitudinal Brasileño sobre el Envejecimiento* (ELSI-Brasil), realizado entre 2015 y 2016 con 4.982 ancianos. El consumo recomendado de frutas y verduras se evaluó en función de preguntas sobre la frecuencia semanal y diaria de frutas, jugos naturales y verduras. La ingesta de cinco o más porciones de estos alimentos en cinco o más días a la semana se consideró como consumo recomendado. Las variables exploratorias incluyeron características sociodemográficas, comportamientos de salud, condiciones de salud y el uso de servicios de salud. La regresión logística univariada y múltiple se utilizó para examinar los factores asociados con el consumo recomendado de frutas y verduras. La prevalencia de la ingesta recomendada de frutas y verduras fue del 12,9% (IC95%: 11,5-14,3). Ese consumo presentó asociaciones con sexo (femenino - OR = 1,40; IC95%: 1,08-1,82), grupo de edad (80 años o más - OR = 1,66; IC95%: 1,16-2,37), escolaridad (8 años o más; OR = 2,07; IC95%: 1,51-2,86), tabaquismo (exfumadores - OR = 0,69; IC95%: 0,55-0,85; y fumadores - OR = 0,50; IC95%: 0,33-0,77) y consultas médicas en los últimos 12 meses (OR = 1,88; IC95%: 1,31-2,71). Los resultados de este estudio mostraron una baja prevalencia del consumo recomendado de frutas y verduras entre los ancianos brasileños, llamando la atención sobre la necesidad de políticas destinadas a aumentar ese consumo en la población estudiada.

## Introduction

The life expectancy of the world population is increasing, which also occurs with the Brazilian population ^1^. In 2021, individuals aged 60 or over represented 14.7% of the Brazilian population ^2^. Recent projections carried out by the Brazilian Institute of Geography and Statistics (IBGE) showed that those aged 65 and over will correspond to 25.5% of the Brazilian population in 2060 ^3^. This population requires special attention, considering that the aging process is accompanied by negative repercussions on health, such as higher prevalence of chronic noncommunicable diseases (NCDs) ^4^.

In Brazil, the Strategic Action Plan to Tackle NCDs proposes actions aimed at the main modifiable risk factors for these diseases, which are smoking, harmful alcohol consumption, physical inactivity, and unhealthy eating ^5^. 

Insufficient consumption of fruits and vegetables is among the top ten risk factors for all-cause mortality ^6^ and is a reality in many countries worldwide ^7^, including Brazil ^8^. On the other hand, the consumption of 400g of fruit and vegetables per day is associated with a reduction in the incidence of cardiovascular diseases and some types of cancer ^9^.

Therefore, the recommended consumption of fruits and vegetables can be used as an indicator of a healthy diet in population studies ^10^ and can be used to verify how this behavior is distributed in the population, thus guiding the actions aimed at the control of NCDs. However, there are few studies analyzing how this marker is distributed in populations of older adults, as reported by a review that addressed the factors related to the consumption of fruits and vegetables in older adults ^11^. Generally, studies that address this topic in Brazil target the general population ^12^ ^,^ ^13^ ^,^ ^14^ ^,^ ^15^ ^,^ ^16^, or are carried out with older adults from specific regions or cities ^17^ ^,^ ^18^, which may not adequately represent the fruit and vegetable consumption of the older Brazilian population as a whole.

Furthermore, most of these studies evaluated predominant sociodemographic aspects and health behaviors ^11^ ^,^ ^18^. However, little is known about the role of variables related to health conditions and the use of health services ^17^. Our findings may help identifying groups that could present an inadequate consumption of fruits and vegetables, to which more specific health actions could be directed. Therefore, this study aimed to estimate the prevalence of recommended consumption of fruits and vegetables and to assess their associated factors in the Brazilian population aged 60 or over.

## Methodology 

### Study population

Baseline data from the *Brazilian Longitudinal Study of Aging* (ELSI-Brazil), conducted between 2015 and 2016 with a national sample representative of the Brazilian population aged 50 years or over, were used. ELSI-Brazil was carried out to investigate the dynamics of aging of the Brazilian population, and analyze its determinants, as well as the social impacts resulting from social and health systems linked to aging. The ELSI-Brazil baseline was conducted with residents from 70 municipalities in the five regions of Brazil. The sample selection was based on strata, which considered the municipality, the census tract, and the household. For smaller municipalities (with up to 750,000 inhabitants), the selection was made in three stages (municipality, census tract, and household) and for larger municipalities, the selection was made in two stages (census tract and household). The final sample was estimated at 10,000 individuals, and 9,412 interviews were carried out, of which 5,432 participants were older adults (≥ 60 years). This study included all older adults (≥ 60 years) who had complete information for all variables of interest, totaling 4,982 individuals (91.7% of the interviewed older adults). All information was obtained from a questionnaire applied in a face-to-face interviews at the participants’ homes, by trained and certified interviewers.

ELSI-Brazil was approved by the Research Ethics Committee of the Oswaldo Cruz Foundation (FIOCRUZ), Minas Gerais (CAAE 34649814.3.0000.5091) and all respondents signed an informed consent form. More details can be found on the research homepage (http://elsi.cpqrr.fiocruz.br/) and in a previous publication ^19^.

### Variables and collection procedures

The outcome variable of this study was the recommended consumption of fruits and vegetables, assessed based on questions about the weekly frequency (number of times per week) and the daily frequency of consumption (number of times per day) of natural fruit juices (whether fruit or fruit pulp or juice concentrate) and of raw or cooked fruits and vegetables (not including potatoes, cassava, or yams).

The recommended consumption of fruits and vegetables was evaluated according to the healthy consumption marker used in the *Risk and Protective Factors Surveillance System for Chronic Non-Comunicable Diseases Through Telephone Interview* (Vigitel), in which participants were classified as having the recommended consumption when the sum of the portions of juices, fruits, and vegetables consumed daily was at least five. Moreover, juices or fruits and vegetables should be consumed on five or more days of the week ^20^, a situation that would correspond to the daily or almost daily consumption of these food ^21^. Due to the difficulties in transmitting the concept of a portion to the interviewee, the number of times per day that natural fruit juice, fruit, or vegetables was consumed was assumed as the number of portions ^20^. The servings consumed were converted into grams according to guidelines developed by the World Health Organization (WHO) with one serving of fruit or vegetables corresponding to 80g ^22^. This marker used by Vigitel was based on the guidelines of the WHO, which recommends a minimum intake of 400g/day of these foods ^9^.

Some authors discuss whether fruit juices should be included when measuring the portions consumed to achieve a recommended daily consumption of fruits and vegetables, since the sugar that is naturally present or that is added to these drinks, may increase the risk of developing a NCDs, such as diabetes mellitus ^23^. However, due to the reduced prevalence of recommended consumption of fruits and vegetables in the Brazilian population verified by other surveys ^20^ ^,^ ^24^, natural fruit juice could be a practical option that could contribute to meeting the recommended daily consumption in this population. Furthermore, fruit juice provides bioactive compounds with antioxidant capacity, which could help preventing NCDs ^25^. Thus, this study considered fruit juice in the assessment of recommended consumption, as seen in other national ^20^ ^,^ ^24^ and international ^7^ publications. However, to ensure the necessary diversification of the diet, the maximum number of juices was limited to one and the maximum number of daily portions computed for fruits or vegetables was limited to three each.

The following exploratory variables were selected based on the literature ^11^: gender (men and women), age group in years old (60-69, 70-79, and 80 or more), years of study (< 4, 4-7 and 8 or more), self-reported skin color (white, black, brown, yellow/indigenous and do not know), marital status (single, married/cohabitation/common-law marriage, divorced/separated, and widowhood), place of residence (urban and rural area), alcohol consumption (no consumption, low-risk consumption, and at-risk consumption), smoking (non-smoker, former smoker, and current smoker), physical activity (insufficient and sufficient), presence of multimorbidity (no and yes) and number of medical consultations in the last 12 months (0, 1-2, and 3 or more).

The alcohol consumption variable was constructed using the report of frequency of weekly consumption and the number of doses consumed using the cut-off points of the United States’ National Institute on Alcohol Abuse and Alcoholism ^26^. The consumption of seven or less doses/week for women and ≤ 14 doses/week for men was considered as low-risk consumption. More than seven doses/week for women and > 14 doses/week for men was considered at-risk consumption. Participants who reported binge drinking (four or more drinks/day for women and five or more drinks/day for men in the last 30 days on a single occasion) were also placed in the at-risk category, regardless of the weekly frequency. For the smoking variable, the classification as current smoker or former smoker was made considering the individual’s answer about the current or past use, respectively, of any tobacco product (industrialized cigarettes, cigars, straw cigarettes), regardless of the amount consumed.

The practice of physical activity was assessed using the *International Physical Activity Questionnaire* that was validated for Brazil ^27^. This questionnaire covered questions about the weekly frequency and duration per day of light, moderate, and vigorous activities. The activities carried out in the week prior to the interview for at least 10 continuous minutes at a time, were considered. Walking at home or to work, as exercising, for leisure or pleasure were considered light activities, while moderate activities included activities such as swimming, cycling, dancing, practicing light aerobic exercises, lifting light weights, and doing housework. Vigorous activities included running, basketball, fast cycling, aerobics, soccer, weightlifting, and heavy household chores. Individuals were considered sufficiently active when they practiced physical activity for 150 minutes/week or more, including walking and activities of moderate or vigorous intensity, time spent doing vigorous activities was doubled ^28^.

Multimorbidity was defined as the presence of two or more chronic diseases ^29^. The following health conditions were considered: high blood pressure, diabetes, heart problems (infarction, angina, and heart failure), stroke, asthma, lung diseases (chronic bronchitis, emphysema, or chronic obstructive pulmonary disease), arthritis or rheumatism, osteoporosis, chronic back problems (back pain, neck pain, low back pain, sciatica, problems with the vertebrae or discs), cancer, chronic kidney failure, depression, Parkinson’s and Alzheimer’s disease, all obtained from a medical diagnosis report. The variable number of medical appointments was analyzed according to the frequency with which the individual visited a physician in the last 12 months. 

### Statistical analysis

A description of the variables was performed for the total sample, as well as a comparison of the percentage distribution of these variables between individuals who had recommended consumption of fruits and vegetables and those who did not, using Pearson’s chi-square test with Rao-Scott correction.

Adjusted and unadjusted logistic regression models were used to obtain the odds ratio (OR) estimate and their respective 95% confidence intervals (95%CI). The goodness of fit of the logistic regression model was evaluated by the Hosmer-Lemeshow test for complex samples ^30^. Furthermore, the presence of multicollinearity between the independent variables was investigated by the variance inflation factor (VIF ≥ 10).

All variables that presented a p-value < 0.05 in the adjusted analysis were associated with the outcome. For the statistical analysis, the Stata program version 14.0 (https://www.stata.com) was used and the weight of the individuals and the effect of the sample design were considered.

## Results

In total, 4,982 older adults participated in this study, with a mean age of 69.7 years (standard deviation = 8.2), ranging from 60 to 105 years. The prevalence of recommended consumption of fruits and vegetables among older Brazilian adults was 12.9% (95%CI: 11.5-14.3).

Table 1 shows the distribution of the characteristics of the study participants for the total sample, according to the recommended consumption of fruits and vegetables. The sample analyzed consisted mainly of women (55.2%), aged from 60 to 69 years (57.7%), with four years of schooling or less (41.5%), self-reported skin color white (43.3%), married, cohabiting or common-law marriage (58.8%), and resident in an urban area (84.2%). Moreover, most participants did not consume alcohol (83%), did not smoke (45.9%), practiced sufficient physical activity (62.7%), had multimorbidity (63%), and had attended to three or more medical appointments in the last 12 months (52.2%). 

**Table 1:** Distribution of characteristics of the study participants in the total sample and according to the recommended consumption of fruits and vegetables. *Brazilian Longitudinal Study of Aging* (ELSI-Brazil), 2015-2016.

Characteristic (%)	Total	Recommended fruits and vegetables consumption *		p-value **
		No	Yes	
Gender				< 0.001
Men	44.8	46.3	34.8	
Women	55.2	53.7	65.2	
Age group (years)				0.037
60-69	57.7	58.5	52.3	
70-79	29.3	29.1	31.2	
80 or more	13.0	12.4	16.5	
Education (years)				< 0.001
< 4	41.5	42.8	32.8	
4-7	30.6	31.1	27.6	
8 or more	27.9	26.1	39.6	
Self-reported skin color				0.532
White	43.3	42.9	45.8	
Black	9.8	9.8	9.6	
Mixed-race	40.5	40.6	39.9	
Yellow/Indigenous	2.8	2.9	2.3	
Do not know	3.6	3.8	2.4	
Marital status				0.241
Single	8.6	8.8	7.6	
Married/Cohabiting/Common-law marriage	58.8	59.3	55.6	
Divorced/Separated	9.7	9.6	10.5	
Widowhood	22.9	22.3	26.4	
Place of residence				0.043
Urban	84.2	83.7	87.7	
Rural	15.8	16.3	12.3	
Alcohol consumption ***				0.111
No consumption	83.0	82.5	86.4	
Low-risk consumption	9.5	9.7	7.8	
At-risk consumption	7.5	7.8	5.8	
Smoking				< 0.001
Non-smoker	45.9	44.0	58.5	
Former smoker	40.1	41.0	33.5	
Current smoker	14.0	15.0	8.0	
Physical activity				0.144
Insufficient	37.3	37.7	34.6	
Sufficient ^#^	62.7	62.3	65.4	
Multimorbidity ^##^				0.911
No	37.0	37.0	37.3	
Yes	63.0	63.0	62.7	
Number of medical consultations in the 12 months prior to the interview				< 0.001
0	14.0	14.8	8.9	
1-2	33.7	34.5	28.2	
3 or more	52.2	50.7	62.9	

* Consumption of five or more servings on five or more days of the week;

** Pearson’s chi-square test with Rao-Scott correction;

*** Low-risk consumption: ≤ 7 doses/week for women or ≤ 14 doses/week for men; at-risk consumption: > 7 doses/week for women or > 14 doses/week for men; also includes those who reported ≥ 4 doses/day for women or ≥ 5 doses/day for men, on a single occasion, in the last 30 days;

^#^ More than 150 minutes/week, including walking and activities of moderate or vigorous intensity;

^##^ Two or more chronic conditions mentioned.

The recommended consumption of fruits and vegetables showed a significant association (p < 0.05) with gender, age group, years of schooling, place of residence, smoking, and number of medical consultations in the last 12 months, in an unadjusted analysis (Table 1).

Table 2 presents the unadjusted and adjusted analysis of the association of sociodemographic characteristics, health behaviors, multimorbidity, and use of health services with the recommended consumption of fruits and vegetables. In the adjusted analysis, the results showed that women had a greater chance of recommended consumption of fruits and vegetables (OR = 1.40; 95%CI: 1.08-1.82), as well as older individuals with 80 years or older (OR = 1.66; 95%CI: 1.16-2.37). Years of schooling was also associated with the consumption of fruits and vegetables, with a greater chance of consumption among those with 8 years or more (OR = 2.07; 95%CI: 1.51-2.86), compared to the group with less than four years of schooling. 

**Table 2:** Unadjusted and adjusted analysis of the association between sociodemographic characteristics, health behaviors, self-reported health condition, and use of health services and the recommended consumption of fruits and vegetables. *Brazilian Longitudinal Study of Aging* (ELSI-Brazil), 2015-2016.

Characteristic	Unadjusted analysis	Adjusted analysis
	OR (95%CI)	OR (95%CI)
Gender		
Men	1.00	1.00
Women	1.61 (1.29-2.01)	1.40 (1.08-1.82)
Age group (years)		
60-69	1.00	1.00
70-79	1.20 (0.93-1.55)	1.29 (0.98-1.71)
80 or more	1.48 (1.07-2.06)	1.66 (1.16-2.37)
Education (years)		
< 4	1.00	1.00
4-7	1.16 (0.82-1.65)	1.19 (0.81-1.74)
8 or more	1.98 (1.51-2.60)	2.07 (1.51-2.86)
Self-reported skin color		
White	1.00	1.00
Black	0.91 (0.59-1.41)	1.10 (0.70-1.72)
Mixed-race	0.92 (0.70-1.21)	1.06 (0.81-1.39)
Yellow/Indigenous people	0.75 (0.42-1.32)	0.85 (0.48-1.51)
Do not know	0.59 (0.29-1.23)	0.76 (0.34-1.73)
Marital status		
Single	1.00	1.00
Married/Cohabiting/Common-law marriage	1.08 (0.71-1.65)	1.21 (0.81-1.81)
Divorced/Separated	1.26 (0.75-2.12)	1.19 (0.72-1.98)
Widowhood	1.36 (0.87-2.13)	1.21 (0.78-1.86)
Place of residence		
Urban	1.00	1.00
Rural	0.72 (0.52-0.99)	0.94 (0.67-1.32)
Alcohol consumption *		
No consumption	1.00	1.00
Low-risk consumption	0.76 (0.54-1.08)	0.80 (0.54-1.17)
At-risk consumption	0.72 (0.47-1.10)	0.91 (0.57-1.44)
Smoking		
Non-smoker	1.00	1.00
Former smoker	0.62 (0.58-0.76)	0.69 (0.55-0.85)
Current smoker	0.40 (0.27-0.60)	0.50 (0.33-0.77)
Physical activity		
Insufficient	1.00	1.00
Sufficient **	1.14 (0.95-1.37)	1.17 (0.97-1.41)
Multimorbidity ***		
No	1.00	1.00
Yes	0.99 (0.80-1.22)	0.80 (0.64-1.01)
Number of medical consultations in the last 12 months		
0	1.00	1.00
1-2	1.37 (0.92-2.02)	1.28 (0.87-1.89)
3 or more	2.07 (1.46-2.94)	1.88 (1.31-2.71)

OR: odds ratio; 95%CI: 95% confidence interval;

Note: OR and 95%CI unadjusted and adjusted for all variables listed in the table, obtained by logistic regression. p-value from Hosmer-Lemeshow test for the adjusted model 0.07.

* Low-risk consumption: ≤ 7 doses/week for women or ≤ 14 doses/week for men; at-risk consumption: > 7 doses/week for women or > 14 doses/week for men; also includes those who reported ≥ 4 doses/day for women or ≥ 5 doses/day for men, on a single occasion, in the last 30 days;

** More than 150 minutes/week, including walking and activities of moderate or vigorous intensity;

*** Two or more chronic conditions mentioned.

A lower chance of presenting the outcome was observed between former smokers (OR = 0.69; 95%CI: 0.55-0.85) and current smokers (OR = 0.50; 95%CI: 0.33-0.77), compared to those who never smoked. The number of medical appointments was associated with the consumption of fruits and vegetables, and those who had three or more consultations (OR = 1.88; 95%CI: 1.31-2.71) in the 12 months prior to the interview were more likely to have recommended consumption of fruits and vegetables. 

## Discussion

Our results showed a low percentage of consumption of fruits and vegetables in the Brazilian population aged 60 years or over, which was more common among older women, more educated, and those who reported having had three or more medical appointments in the 12 months prior to the interview. On the other hand, smokers and former smokers reported lower consumption of fruits and vegetables, compared to the group that had never smoked.

The low percentage of recommended consumption of fruits and vegetables observed in this present study (12.9%) was lower than that reported in a study conducted with older adults residing in Goiânia, Goiás State (16.6%) ^17^. The authors of this study only evaluated the frequency of consumption of fruit and/or fruit juice, raw leafy vegetables, and cooked legumes. The outcome was the consumption of the three food groups at least once per day ^17^ without evaluating the number of servings. Therefore, some older adults could have a consumption lower than the 400g/day recommended by the WHO ^9^. On the other hand, data from Vigitel conducted in 2021, which evaluated the outcome in a similar way to that evaluated in this article, showed that about 24.8% of those aged 55 and over reached the recommended consumption of fruits and vegetables ^20^, higher than that observed in our study. 

Other studies conducted in developing ^31^ and developed countries ^32^ ^,^ ^33^ also found prevalence higher than those presented by ELSI-Brazil. For example, a study conducted using data from the *World Health Survey* (2002-2003) in 52 low- and middle-income countries found that about 22% of the adults and older adults assessed had adequate consumption of fruits and vegetables ^7^. In this international study, unlike ELSI-Brasil, which considered a portion as having 80g, cards with different standard portions according to the specificities of each country were presented to the interviewees, seeking to better quantify the intake of fruits and vegetables.

The variation between the prevalence of recommended consumption of fruits and vegetables among the studies evaluated, may be related to the different consumption profiles of the populations. However, part of this difference can be attributed to the different ways of measuring the outcome ^7^. For example, the *Brazilian National Health Survey* (PNS) conducted in 2013 ^34^ found a higher prevalence of recommended consumption of fruits and vegetables among older adults when compared to the survey conducted in 2019 ^8^ (40.1 versus 17.9, respectively). This difference may be related to the change in the way of evaluating intake and of classifying the recommended consumption between surveys. In 2013, the PNS used two separate questions to assess the consumption of raw and cooked vegetables, with respondents being able to answer that they ate each of these food groups up to three times per day, totaling the intake of up to six daily servings. In 2019, the two questions were unified, and the participant could only report the consumption of up to three servings of raw and cooked vegetables per day. In addition, the 2019 PNS considers the recommended consumption of vegetables or fruits (including juice) to be 25 or more times per week. This definition differs from the one adopted in the 2013 PNS, where the recommended consumption of these foods was considered to be five or more times per day, with the minimum consumption being: one fruit and one type of salad or one cooked vegetable per day. In this way, we can verify that the different ways of evaluating the outcome, the response options for the number of servings consumed, the evaluation of different serving sizes or the use of a standard portion, the inclusion or not of natural fruit juice in the sum of servings, are issues that may interfere with the under or overestimation of the prevalence of recommended fruits and vegetables intake and, consequently, the factors associated with this outcome.

Due to these differences, which jeopardize the comparability among studies, there is a need to standardize the way of assessing the recommended consumption of fruits and vegetables, which would allow a more robust assessment of this indicator. Even so, one can perceive a low consumption of fruits and vegetables among older Brazilian adults, similar to what was observed in other populations. Issues related to the low quality and variety of products offered by local businesses ^35^, high cost, and lack of habit ^36^ have been identified as important barriers to the consumption of these foods. Also, the increase in the participation of ultra-processed food in the Brazilian diet, observed in the last decades, contributes to the low consumption of fruits and vegetables in the Brazilian population ^37^.

Regarding the factors associated with the recommended consumption of fruits and vegetables, gender played an important role, with women having a greater chance of consumption, compared to men, even after adjusting for the other sociodemographic variables. This result was consistent with other studies carried out with representative samples of the Brazilian population ^8^ ^,^ ^24^, with regional ^17^ and international studies ^11^ ^,^ ^33^, conducted with older adults. On the other hand, the longitudinal study EpiFloripa Aging did not find differences in the recommended consumption of fruits and vegetables between older men and women ^18^. The difference in consumption between the genders can be attributed to a greater nutritional knowledge by women ^38^. In addition, women seek health services more frequently than men ^39^, which may be related to greater access to information on healthy eating, and thus, contributing to an adequate consumption of fruits and vegetables.

A greater chance of recommended consumption of fruits and vegetables was also observed among older individuals (80 years or more), compared to the 60-69 age group. This trend was also observed in other surveys conducted with Brazilian adults and older adults ^8^ ^,^ ^20^ ^,^ ^24^, as well as with only older adults ^40^. This finding may be related to health awareness acquired throughout life, which may be due to greater access to information on healthy eating due to a higher number of medical appointments ^8^. Furthermore, there may also be an improvement in their diets to prevent or manage chronic diseases, which are more prevalent in older adults ^4^. In addition, this group was able to experience the period before the food transition, when there was a different availability of fruits and vegetables and ultra-processed foods, which could also justify a higher consumption of fruits and vegetables in this population, due to the cohort effect, as already suggested by other authors ^41^. On the other hand, some international studies have found that with increasing age there was a decrease in consumption of fruits and vegetables ^7^ ^,^ ^33^ or did not verify an association between age and the recommended consumption of fruits and vegetables ^18^. In addition to the methodological differences between the studies, we hypothesized that the cultural differences between countries could explain the contradictory results related to age and consumption of fruits and vegetables.

ELSI-Brazil participants with more years of schooling were more likely to have a recommended consumption of fruits and vegetables, which is consistent with the results of other studies conducted in different populations of older adults ^11^ ^,^ ^40^. This fact can be attributed, at least partly, to the higher family income of this group with a consequent increase in purchasing power ^42^. This hypothesis can be reinforced by the results of a study that evaluated the data from the *Brazilian Household Budget Survey* (POF), conducted from 2008 to 2009 and from 2017 to 2018. The authors found that the percentage of purchase of fruits and vegetables was higher among those with higher income and that the households with lower income also had less variety of these foods in their purchases ^43^. Additionally, it should also be noted that individuals with more years of schooling have greater access to information, greater nutritional knowledge and can thus make healthier food choices ^11^.

Regarding health behaviors, the results of this study showed a lower consumption of fruits and vegetables among individuals who smoked at the time of the interview and former smokers. These results are consistent with previous studies ^44^ ^,^ ^45^ demonstrating an agglutination of behaviors that characterize a healthy lifestyle, such as the consumption of fruits and not smoking. Thus, these findings demonstrate the importance of planning joint actions to increase the fruits and vegetables consumption since a healthy habit can potentially influence others.

Among the indicators of health conditions and service use, a higher number of medical consultations was associated with a greater chance of presenting recommended fruits and vegetables consumption. Generally, individuals with many morbidities tend to have more medical consultations to control these conditions ^46^, and thus can receive information about healthy eating during these consultations. However, studies involving this subject are scarce ^47^ and have not evaluated only older adults. Therefore, although our results show the potential of consultations to influence the fruits and vegetables consumption, considering the observed association, regardless of the other factors explored in this investigation, this association still needs to be further explored.

This study has some limitations that must be considered when interpreting the results presented. First, it is a cross-sectional study that does not allow us to establish temporal relationships among the variables, although we only aimed to analyze the factors associated with the outcome. Second, we measured the outcome using markers that assessed the frequency of consumption, replacing the use of other instruments such as food frequency questionnaires. Furthermore, ELSI-Brazil, as well as other Brazilian studies ^8^ ^,^ ^20^, considers the daily frequency of consumption as the number of servings, which provides less accurate information about the quantities intake ^48^, impacting in the classification of those with adequate consumption and in the association between the explanatory variables and this outcome. 

Nevertheless, evaluating the consumption of fruits and vegetables, using consumption markers has been very common in other surveys ^20^ ^,^ ^24^, as it is a simple instrument with a fast application, which aims to identify inadequacies in food consumption, contributing to the knowledge of dietary practices in population studies ^49^. Finally, we considered one serving of juice in the total number of servings consumed when assessing the recommended fruits and vegetables consumption. However, although some authors question this inclusion, the convenience of consuming natural fruit juice is an easier alternative to reach the daily recommendation for fruits and vegetables among older adults ^25^, especially among those with compromised oral and dental health ^11^. Despite these limitations, this was the first Brazilian study that evaluated the factors associated with the fruits and vegetables consumption in a representative sample of older adults, contributing to the knowledge about this subject in this population. Moreover, our findings provide important information to support actions aiming at promoting adequate and healthy food consumption at the national level, also promoting health and guaranteeing the human right to adequate food among older Brazilians. These actions must be aligned with the economic and social context of this population and must respect and value the cultural dimensions of food. Furthermore, the study used standardized procedures and trained technicians to collect information, which ensured its internal validity, thus producing quality data.

## Conclusion

Our results showed a low consumption of fruits and vegetables among Brazilians aged 60 or over and that sociodemographic, behavioral and health service use factors influenced this consumption, enabling the identification of the groups with the greatest chance of meeting the recommended consumption of these foods. 

Despite advances in the Brazilian National Food and Nutrition Policy, especially regarding actions to promote adequate and healthy food consumption, such as the publication of the *Dietary Guidelines for the Brazilian Population* ^50^ and the protocols for the dietary guidelines for Brazilian population used in nutrition advising for the older population ^51^, the low fruits and vegetables consumption in the older Brazilian population still remains. These results demonstrate the need to strengthen public policies and existing programs in food and nutrition, which promote the recommended fruits and vegetables consumption among older individuals since the prevention and control of diseases that affect these individuals is essential. 

This scenario highlights the need to implement new strategies, such as the “5-a-day” program, a public health campaign adopted by different countries ^52^ that seeks to guarantee the daily intake of five servings of fruit and vegetables. The strategy should be aimed mainly at the most vulnerable groups, that is, men, younger individuals, less educated, smokers, and who have fewer medical consultations. Besides educational strategies, market regulation actions that provide opportunities for the fruits and vegetables production and distribution, such as the Food Acquisition Program and the inclusion of open-air markets, should be prioritized. These actions could help resolve issues such as the disparity in financial and physical access to these foods.

## References

[1] World Health Organization (2016). Life expectancy increased by 5 years since 2000, but health inequalities
persist.

[2] Instituto Brasileiro de Geografia e Estatística Pesquisa Nacional por Amostra de Domicílios Contínua: características
gerais dos moradores 2020-2021..

[3] Instituto Brasileiro de Geografia e Estatística (2018). Projeção da população 2018: número de habitantes do país deve
parar de crescer em 2047.. Agência IBGE de Notícias.

[4] World Health Organization (2011). US National Institute of Aging. Global health and aging..

[5] Departamento de Análise em Saúde e Vigilância de Doenças Não
Transmissíveis, Secretaria de Vigilância em Saúde, Ministério da
Saúde Plano de ações estratégicas para o enfrentamento das doenças crônicas e
agravos não transmissíveis no Brasil 2021-2030..

[6] World Heallth Organization (2009). Global health risks: mortality and burden of disease attributable to
selected major risks.

[7] Hall JN, Moore S, Harper SB, Lynch JW (2009). Global variability in fruit and vegetable
consumption.. Am J Prev Med.

[8] Ministério da Saúde (2020). Pesquisa Nacional de Saúde 2019: percepção do estado de saúde, estilos
de vida, doenças crônicas e saúde bucal. Brasil e grandes regiões.

[9] World Health Organization (2003). Diet, nutrition and the prevention of chronic diseases: report of a
joint WHO/FAO expert consulation.

[10] Monteiro CA, Moura EC, Jaime PC, Claro RM (2008). Validity of food and beverage intake data obtained by telephone
survey. Rev Saúde Pública.

[11] Nicklett EJ, Kadell AR (2013). Fruit and vegetable intake among older adults a scoping
review. Maturitas.

[12] Damiani TF, Pereira LP, Ferreira MG (2015). Consumo de frutas, legumes e verduras na Região Centro-Oeste do
Brasil: prevalência e fatores associados.. Ciênc Saúde Colet.

[13] Figueiredo ICR, Jaime FPC, Monteiro CA (2008). Fatores associados ao consumo de frutas, legumes e verduras em
adultos da cidade de São Paulo. Rev Saúde Pública.

[14] Neutzling MB, Rombaldi AJ, Azevedo MR, Hallal PC (2009). Fatores associados ao consumo de frutas, legumes e verduras em
adultos de uma cidade no Sul do Brasil. Cad Saúde Pública.

[15] Campos VC, Bastos JL, Gauche H, Boing AF, Assis MAA (2010). Fatores associados ao consumo adequado de frutas, legumes e
verduras em adultos de Florianópolis. Rev Bras Epidemiol.

[16] Jaime PC, Figueiredo ICR, Moura EC, Malta DC (2009). Fatores associados ao consumo de frutas e hortaliças no Brasil,
2006. Rev Saúde Pública.

[17] Silveira EA, Martins BB, Abreu LRS, Cardoso CKS (2015). Low consumption of fruit, vegetables and greens associated
factors among the elderly in a Midwest Brazilian city. Ciênc Saúde Colet.

[18] Souza BB, Cembranel F, Hallal ALC, d'Orsi E (2019). Consumption of fruits and vegetables and association with life
habits and nutritional status a prospective study in a cohort of the
elderly. Ciênc Saúde Colet.

[19] Lima-Costa MF (2018). Aging and public health the Brazilian Longitudinal Study of Aging
(ELSI-Brazil). Rev Saúde Pública.

[20] Departamento de Análise em Saúde e Vigilância de Doenças Não
Transmissíveis.Secretaria de Vigilância em Saúde.Ministério da
Saúde (2021). Vigitel Brasil 2021: vigilância de fatores de risco e proteção para
doenças crônicas por inquérito telefônico. Estimativas sobre frequência e
distribuição sociodemográfica de fatores de risco e proteção para doenças
crônicas nas capitais dos 26 estados brasileiros e no Distrito Federal em
2021.

[21] Monteiro CA, Moura EC, Jaime PC, Caro RM (2008). Validade de indicadores do consumo de alimentos e bebidas obtidos
por inquérito telefônico. Rev Saúde Pública.

[22] Food and Agriculture Organization (2003). What is a serving?.

[23] Muraki I, Imamura F, Manson J, Hu F, Willet W, Van Dan RM (2013). Fruit consumption and risk of type 2 diabetes results from three
prospective longitudinal cohort studies. BMJ.

[24] Jaime PC, Stopa SR, Oliveira TP, Vieira ML, Szwarcwald CL, Malta DC (2015). Prevalence and sociodemographic distribution of healthy eating
markers, National Health Survey, Brazil 2013. Epidemiol Serv Saúde.

[25] Benton D, Young HA (2019). Role of fruit juice in achieving the 5-a-day recommendation for
fruit and vegetable intake. Nutr Rev.

[26] National Institute on Alcohol Abuse and Alcoholism Rethinking drinking: alcohol and your health..

[27] Matsudo S, Araújo T, Matsudo V, Andrade D, Andrade E, Oliveira LC (2001). International Physical Activity Questionnaire (IPAQ) study of
validity and reliability in Brazil. Rev Bras Ativ Fís Saúde.

[28] World Health Organization (2010). Global recommendations on physical activity for health.

[29] World Health Organization Multimorbidity: technical series on safer primary care..

[30] Archer KJ, Lemeshow S, Hosmer DW (2007). Goodness-of-fit tests for logistic regression models when data
are collected using a complex sampling design. Comput Stat Data Anal.

[31] Peltzer K, Phaswana-Mafuya N (2012). Fruit and vegetable intake and associated factors in olders
adults in South Africa.. Glob Health Action.

[32] Appleton KM, McGill R, Woodside V (2009). Fruit and vegetable consumption in older individuals in Northern
Ireland levels and patterns. Br J Nutr.

[33] Osborne B, Cooper V Health Survey for England 2017: Adult heath related behaviours..

[34] Instituto Brasileiro de Geografia e Estatística Pesquisa Nacional de Saúde 2013: percepção do estado de saúde, estilos
de vida e doenças crônicas..

[35] Figueira TR, Lopes ACS, Modena CM (2016). Promoters and barriers to fruit and vegetable consumption among
Health Academy Program's users. Rev Nutr.

[36] Santos GMC, Silvia AMR, Loch MR, Carvalho W, Rech CR (2019). Perceived barriers for the consumption of fruits and vegetables
in Brazilian adults. Ciênc Saúde Colet.

[37] Levy RB, Andrade GC, Cruz GL, Rauber F, Louzada MLC, Claro RM (2022). Three decades of household food availability according to NOVA -
Brazil, 1987-2018. Rev Saúde Pública.

[38] Baker AH, Wardle J (2003). Sex differences in fruit and vegetable intake in older
adults. Appetite.

[39] Silva SLA, Torres JL, Peixoto SV (2020). Factors associated with preventive health services search among
Brazilian adults National Health Survey, 2013. Ciênc Saúde Colet.

[40] Eustaquio C, Druesne-Pecollo N, Latino-Martel P, Dauchet L, Hercberg S, Bertrais S (2008). Socioeconomic differences in fruit and vegetable consumption
among middle-aged French adults: adherence to the 5 a day
recommendation.. J Am Diet Assoc.

[41] Costa GAM, Lopes MS, Lopes ACS (2022). Fruit and vegetable consumption across generations of primary
Brazilian care users.. Nutrition.

[42] Claro RM, Carmo HC, Machado FM, Monteiro CA (2007). Income, food prices, and participation of fruit and vegetables in
the diet. Rev Saúde Pública.

[43] Oliveira N, Santin F, Paraizo TR, Sampaio JP, Moura-Nunes N, Canella DS (2021). Lack of variety of fruit and vegetables available in Brazilian
households: data from the Household Budget Surveys of 2008-2009 and
2017-2018.. Ciênc Saúde Colet.

[44] Chou KL (2008). The prevalence and clustering of four major lifestyle risk
factors in Hong Kong Chinese older adults. J Aging Health.

[45] Mello GT, Silva KS, Costa BG, Borgatto AF (2019). Patterns of risk behaviors in Brazilian older adults A latent
class analysis. Geriatr Gerontol Int.

[46] Malta DC, Bernal RTI, Lima MG, Araújo SSC, Silva MMA, Freitas MIF (2017). Noncommunicable diseases and the use of health services analysis
of the National Health Survey in Brazil. Rev Saúde Pública.

[47] Lima-Costa MF, Loyola FAI (2008). Associated factors to the use and the satisfaction with heath
services among National Health System users in the Metropolitan Region of
Belo Horizonte, state of Minas Gerais, Brazil. Epidemiol Serv Saúde.

[48] Pereira RA, Sichieri R, Kac G, Sichieri R, and Gigante DP (2007). Epidemiologia nutricional.

[49] Departamento de Atenção Básica.Secretaria de Atenção à
Saúde.Ministério da Saúde (2015). Orientações para avaliação de marcadores de consumo alimentar na atenção
básica.

[50] Departamento de Atenção Básica, Secretaria de Atenção à Saúde,
Ministério da Saúde (2014). Guia alimentar para a população brasileira..

[51] Ministério da Saúde Fascículo 2 - protocolos de uso do guia alimentar para a população
brasileira na orientação alimentar da população idosa..

[52] Castiglione C, Mazzocchi M (2019). Ten years of five-a-day policy in the UK nutritional outcomes and
environmental effects. Ecol Econ.

